# Necrotizing Epididymo-Orchitis: A Rare Manifestation of COVID-19

**DOI:** 10.1155/2022/1891429

**Published:** 2022-07-01

**Authors:** Ali Tavoosian, Sana Ahmadi, Seyed Mohammad Kazem Aghamir

**Affiliations:** Urology Research Center, Tehran University of Medical Sciences, Tehran, Iran

## Abstract

Epididymo-orchitis is an infection of the epididymis and testis, one of the most common urogenital infections. It can be seen at any age. It is caused by sexually transmitted microorganisms and nonsexual transmitted pathogens. Viruses such as mumps and cytomegalovirus can also cause epididymo-orchitis. During the COVID-19 pandemic, in case of abnormal clinical manifestations of COVID infection and inadequate therapeutic response to the routine therapies, this disease with unusual manifestations should be considered. The case introduced in this paper is a 55-year-old man referred to a urology clinic with typical clinical presentations of epididymo-orchitis. Diagnosis by color Doppler examination and ultrasound also confirmed epididymo-orchitis. The patient underwent appropriate and routine treatment for epididymo-orchitis. Because of the lack of adequate clinical response and the continuation of fever and the development of scrotal lesions and the results of the control ultrasound, which suggested rupture of the tunica albuginea capsule, he underwent surgical exploration and subsequent orchiectomy. Due to the unconventional conditions and the usual culture and pathology, COVID-19 PCR was also performed on the tissues. The PCR showed tissue infection with COVID-19. The patient's clinical condition improved with an orchiectomy, the fever stopped, and he was discharged in a good general condition. It should be noted that before referral to the urology clinic and during hospitalization, evaluation, and treatment, the patient had no evidence in favor of respiratory tract infection with the coronavirus.

## 1. Introduction

In the last days of 2019, coronavirus disease 2019 (COVID-19), first identified in China, became a worldwide pandemic. Respiratory compromise is the most important feature of the disease. Soon, the COVID-19 outbreak became a world challenge [1, 2]. The common symptoms of COVID-19 are cough, fever, and fatigue [3].

Epididymo-orchitis is a common urological condition that occurs at any age. The most common cause of epididymo-orchitis in young adults is sexually transmitted pathogens, such as Chlamydia trachomatis and Neisseria gonorrhoeae. In older men, epididymo-orchitis is most often caused by nonsexually transmitted Gram-negative bacteria causing urinary tract infections, which rarely occur without concomitant epididymitis [4–6]. Orchitis, especially in young males, can be caused by a virus such as mumps (which is the most common cause of viral orchitis), rubella, coxsackievirus, varicella, echovirus, and cytomegalovirus. Widespread MMR vaccination decreased the frequency of mumps orchitis [4, 5].

During the past two years of COVID-19 pandemic, many presentations and complications such as several genitourinary disorders have been reported worldwide [5, 7].

## 2. Case Presentation

A 55-year-old gentleman was referred to a urologist due to the right hemiscrotal pain and swelling four days ago, and fever started two days ago. He reported some irritative symptoms of the lower urinary tract such as dysuria and frequency. There was no obstructive symptom. There was no history of unprotected sexual contact. He agreed on orchiectomy and report his case by signing the written informed consent, and the case report is based on CARE guideline. On initial examination, 101.3°F (38.5°C) oral fever, erythema of right hemiscrotal skin was founded, and the skin of the right hemiscrotal was edematous. We found no evidence of a sexually transmitted disease (such as purulent discharge) on examination. On palpation, enlargement of the right testicle and stiffness and induration of the right epididymis were evident. On touch, the testicle had tenderness, which, based on an examination, was suggested to be a right epididymo-orchitis.

**Figure 1:**
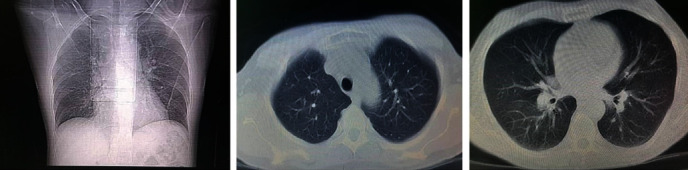
(a) First sonographic image. (b) Second sonographic images.

**Figure 2:**
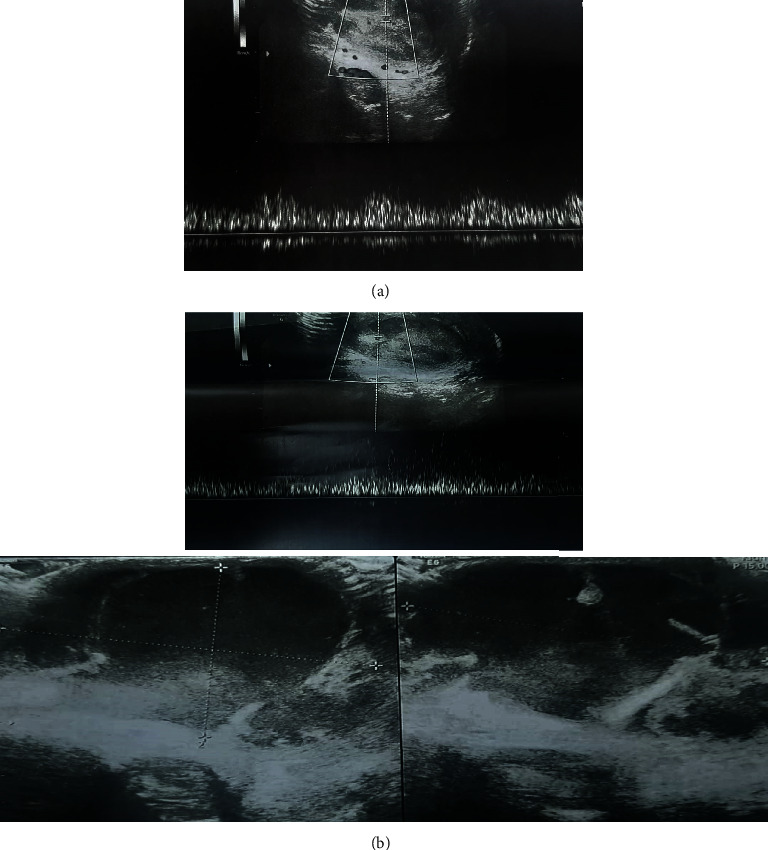
(a) Chest CT and (b) Sonography report.

In color Doppler ultrasound performed from the testis (Figure 1), the left testicle was normal, the skin of the right scrotum was edematous, with increasing the size of the right testicle and its echo was heterogeneous and decreased. An increase in the size of the right epididymis and an increase in the arterial flow of the right epididymis and testis, along with mild hydrocele with internal debris, have also been reported. The patient had no history of medication, and the patient's complete history indicates no illness and past medical problems and no history of unsafe sex. Also, he had no history of exposure to the COVID-19 for more than his last two weeks. His complete blood count (CBC) to evaluate his overall health detects WBC: 9200, neutrophil: 54, lymphocyte: 36, RBC: 10-12, BAC: rare, and negative nitrite. His chest CT-scan images are presented in Figure 2. The above items in the ultrasound report confirmed the right epididymo-orchitis. With the mentioned diagnosis, the patient is admitted to the urology department and underwent the following treatment:

1. Complete bed rest
2. Ciprofloxacin 400 mg IV/BID
3. Tablet of Ibuprofen 400 mg PO/TDS
4. Testis elevation
5. Testis cold compression

Despite 48 hours of intravenous antibiotic treatment, the patient's fever continued, and the scrotal swelling increased sharply. The scrotal skin was severely edematous on the second ultrasound (Figure 1), and severe hydrocele was reported in the right hemiscrotal with no abscess formation. The right testicle continues to increase in size, and its echo has heterogeneity and increased vascular flow.

The patient did not have any upper respiratory symptoms before referring to the urology clinic with a mentioned urological manifestation and during the hospital stay. Due to the persistence of fever and the consult with an infectious disease specialist, a request was made for nasopharyngeal secretions tests and high-resolution computed tomography (HRCT) of the chest to rollout the COVID-19. There was no change in the patient's IV antibiotic regimen. HRCT showed no evidence of COVID 19-induced pulmonary involvement, and the nasopharyngeal test was negative for COVID19. During the 96 hours since admission, the patient remained feverish. The testicular pain increased sharply. On examination of the right hemiscrotal, tenderness, severe scrotal skin edema, and severe hydrocele were evident. The patient underwent color Doppler ultrasound of the testicles again (Figure 1), ruptured of the tunica albuginea capsule, and exudation of the intratesticular tissues to outside the tunica albuginea capsule was reported.

The patient was a candidate for exploratory surgery. By transverse incision on the right hemiscrotal, we entered the space between the tunica-vaginalis and the tunica-albuginea, and about 800 ml of fluid (which was pyocele) was drained. Extensive rupture of the tunica albuginea in the middle bridge of the right testicle was evident, along with extensive necrosis of the internal tissues of the right testicle. The decision was made for a right orchiectomy. Extracted tissues were sent for pathology, culture, antibiogram, and PCR of testicular tissue for COVID-19. After orchiectomy, the patient's general condition has improved. And within 12 hours of surgery, the patient's fever stops. We ordered PCR test of the testicle tissue. Twenty-four hours after the tests, PCR of the testicular tissue submitted was positive for COVID 19.

The patient was treated with remdesivir based on the infectious disease specialist recommendation for five days based on infectious counseling. The patient had no upper or lower respiratory tract infection symptoms during this time. Finally, the patient was discharged in a good general condition without fever and significantly reduced swelling and scrotal edema.

## 3. Discussion

Although COVID-19 in some patients can be presented without fever, and many of the patients did not have abnormal radiological findings, other rare results were found in some of them, such as urogenital signs and symptoms [1, 7]. Urogenital involvement of coronavirus is rare and limited with some case reports [4, 8, 9]. Some of the urogenital complications caused by COVID-19 have been secondary to hypercoagulable states [5]. There are some case reports regarding patients with epididymo-orchitis symptoms such as abdominal, testicular, and back pain; bilateral testicular warmth, testicular discomfort, swelling, pain, and erythema; and concurrent COVID-19, without any respiratory symptom [5, 6, 10–13]. These studies reported NSAID or antibiotic therapy would be successful in all patients with testicular pain, and no patient required more invasive procedures [4, 6, 14, 15].

In our study, we describe a case of a gentleman with no symptom of coronavirus disease and negative respiratory tests for COVID-19, who was referred to our clinic with epididymo-orchitis symptoms. The question is how can the coronavirus infect the testicles?

The mechanism of the COVID-19 infection is binding to Angiotensin-Converting Enzyme 2 (ACE2). This is known as the main pathway of influencing host cells [16]. ACE2 is expressed in many tissues in the genitourinary system. Today, we know that the testis presents some conditions that might favor COVID-19 infection, such as the expression of ACE2, and the fact that Leydig cells of the testis receptors can act as the ACE2 receptor. This finding could explain the COVID-19 passage into the testicular microcirculation where reduced blood flow and presence of its receptor (ACE2) could enhance testicular infection [5, 13, 17–21].

## 4. Conclusion

This paper presents a case of epididymo-orchitis who was infected by a coronavirus. We also tried to answer the question of how the testicles become infected with the coronavirus by perusing other similar cases presented all around the world. Further studies are needed to determine exactly how the coronavirus infects the genitourinary tract and what the effects of the infection will be in the future.

## Data Availability

Data will be provided on request.
